# SKN-1/Nrf, A New Unfolded Protein Response Factor?

**DOI:** 10.1371/journal.pgen.1003827

**Published:** 2013-09-12

**Authors:** Keith P. Choe, Chi K. Leung

**Affiliations:** Department of Biology and Genetics Institute, University of Florida, Gainesville, Florida, United States of America; The University of Texas Health Science Center at Houston, United States of America

Cell function requires simultaneous regulation of numerous processes, often under variable conditions. Several inducible pathways have been defined as being responsible for maintaining homeostasis under the threat of a particular stress such as heat, oxidizing conditions, exposure to pathogens, or loss of proteostasis. The challenge now is to understand how and why these pathways interact in basal, stress, and pathological states. The *Caenorhabditis elegans* inducible transcription factor SKN-1, a homolog of mammalian Nrf proteins, has been defined as the transcription factor that responds to oxidative stress. In this issue of *PLOS Genetics*, Glover-Cutter et al. [1] challenge this paradigm by showing that SKN-1 directly regulates the genes of core regulators and effectors of the endoplasmic reticulum (ER) unfolded protein response (UPR) and that the UPR plays a role in activation of the antioxidant/detoxification response.

**Figure 1:**
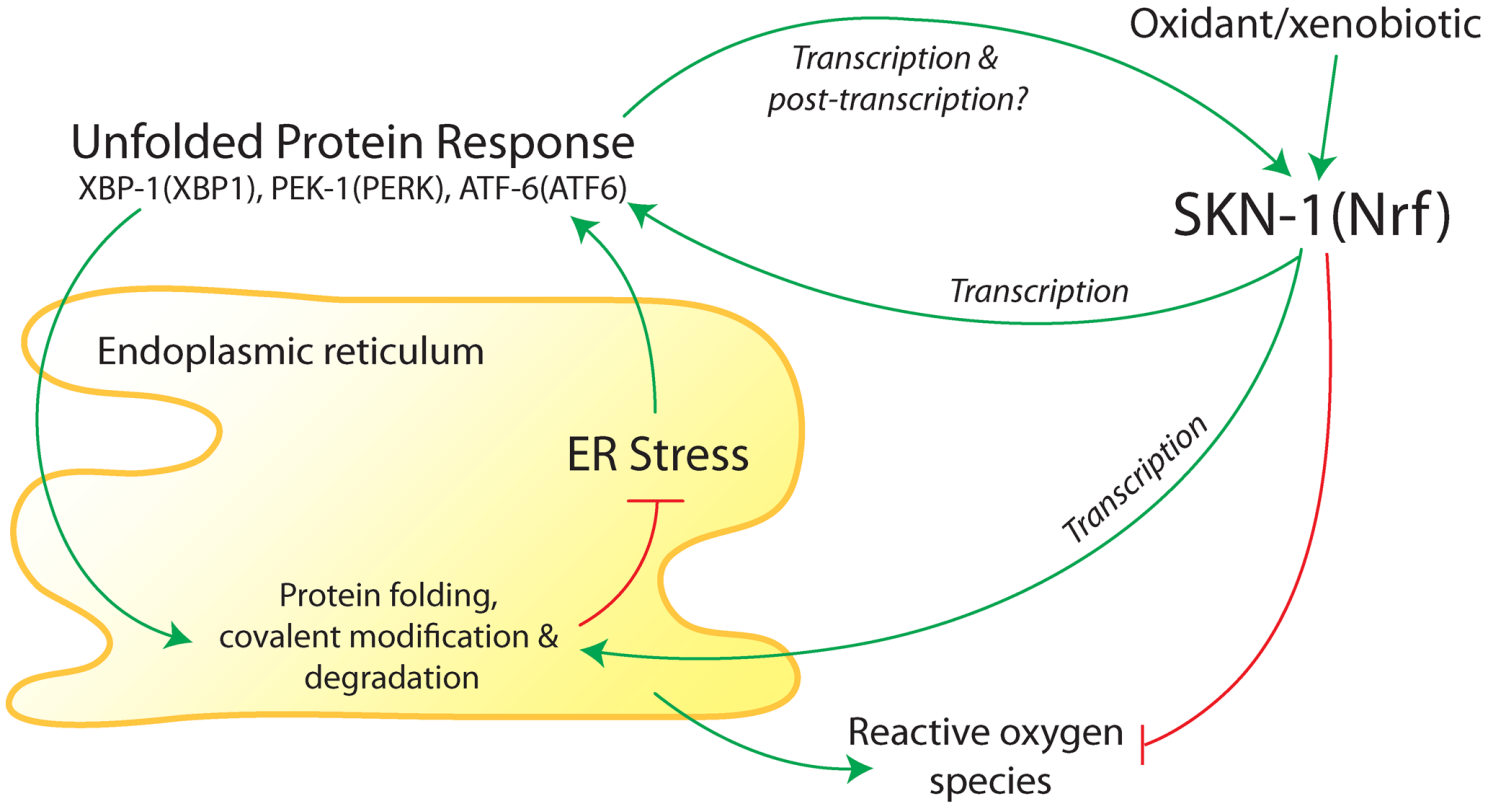
Summary of interactions between the endoplasmic reticulum (ER) unfolded protein response (UPR) and antioxidant/detoxification transcription factor SKN-1 in *C. elegans*. The ER folds and modifies newly synthesized membrane and secreted proteins. Accumulation of misfolded proteins in the ER activates three canonical branches of the UPR, which are mediated by XBP-1, PEK-1, and ATF-6 (mammalian homologs in parentheses). Transcriptional targets of the UPR function to promote protein folding and covalent modification of new proteins and degradation of misfolded proteins. SKN-1 (homolog of mammalian proteins Nrf1, Nrf2, and Nrf3) was found to transcriptionally regulate core UPR regulators and some downstream targets during ER stress [1]. Core UPR regulators also transcriptionally regulate SKN-1 during ER stress and oxidative/xenobiotic stress [1]. Based on studies in mammalian cells [12], [13], SKN-1 would also be expected to buffer reactive oxygen species that are produced in the ER. A long variant of SKN-1 may reside in the ER membrane (not shown). Regulation of SKN-1 during ER and oxidative/xenobiotic stress may include post-translational modifications.

ER homeostasis requires coordination of protein translation, folding, and covalent modification; availability of energy and substrates; and maintenance of a redox environment that is suitable for disulfide bond formation. Disruption of ER homeostasis can cause accumulation of misfolded proteins in the ER lumen, referred to as “ER stress,” which can impair cell function and eventually trigger cell death [2]–[4]. The UPR is a eukaryotic signaling program that responds to ER stress by inhibiting protein translation, inducing protein folding chaperones, and directing the degradation of misfolded proteins [2]–[4] (Figure 1). Redox homeostasis in animal cells is controlled in part by a family of transcription factors represented by SKN-1 in *C. elegans* and Nrf1, Nrf2, and Nrf3 in mammals (Figure 1). SKN-1 and Nrf2 have well-established roles in promoting redox homeostasis and small molecule detoxification, and SKN-1 has been shown to promote longevity [5]–[8].

Glover-Cutter et al. [1] conducted an extensive series of genetic and molecular experiments to investigate regulatory interactions between SKN-1 and the UPR; many of their findings are summarized in Figure 1. They showed that SKN-1 regulates numerous genes involved in ER function during ER stress that are not typically activated by SKN-1 during oxidative stress. These include protein chaperones and homologs of the following core UPR components: BiP (an unfolded protein sensor), PERK (a protein kinase), IRE1 (a protein kinase and mRNA endonuclease), and the transcription factors XBP1, ATF4, and ATF6. During ER stress, SKN-1 protein was shown to associate with loci for homologs of ATF4, ATF6, XBP1, and IRE1, indicating that regulation of core UPR genes by SKN-1 is likely to be direct.

How is SKN-1 activated by ER stress? The authors observed elevated *skn-1* mRNA and protein levels during ER stress [1]. Processing of protein disulfide bonds in the ER can elevate reactive oxygen species (ROS) [4], [9], a well-established stimulus for SKN-1 that could simply activate it secondarily. However, the authors demonstrated induction of *skn-1* mRNA by a strong reducing agent and by silencing of the worm ER oxidoreductase, conditions that cause ER stress and decrease ROS [1]. Furthermore, the *C. elegans* homologs of XBP1 and ATF6, and SKN-1 itself, all associated with the *skn-1* locus during ER stress and were found to play a role in induction of *skn-1* mRNA [1]. Therefore, activation of SKN-1 during ER stress appears to be at least partly transcriptional *via* UPR transcription factors.

If SKN-1 is required for the UPR, then could the UPR also be required for the antioxidant/detoxification response? The answer may be yes. During oxidative stress, core components of the UPR were required for induction of *skn-1* mRNA and some SKN-1 target genes. It is of additional interest that activation of p38 MAPK by phosphorylation, which activates SKN-1 under conditions of oxidative stress, also required components of the UPR [10].

Important regulatory and functional interactions between the UPR and Nrf2 were previously known for mammalian cells [4], [11]–[13]. Nrf2 is phosphorylated and activated by PERK during ER stress and promotes cell survival by maintaining redox homeostasis together with ATF4 [12]–[13]. Nrf2 also activates expression of proteasome subunits and may support degradation of misfolded proteins during ER stress [11]. So what is different about the current findings? Transcriptional regulation of core UPR transcription factors and downstream effectors by SKN-1 is a far more central function in the ER stress response than has previously been reported for SKN-1/Nrf family members. Details of the molecular interactions may not all be conserved, but the findings for SKN-1 raise the possibility that Nrf1, Nrf2, or Nrf3 may be centrally integrated into the mammalian UPR.

As with any new findings, important new questions follow. SKN-1 was shown to contribute to survival of ER stress *in vivo* [1]. Determining the relative importance of SKN-1–mediated UPR gene regulation versus redox homeostasis would be challenging, but is needed to understand the function of these newly identified regulatory interactions. Other than PERK phosphorylation of Nrf2 [12]–[13], little is known about post-translational regulation of SKN-1/Nrf proteins during ER stress. Evidence was provided for association of a long SKN-1 variant with the ER [1], and Nrf1 and Nrf3 each have a predicted transmembrane domain and have been reported to be associated with the ER membrane [14]–[16]. In unstressed cells, ATF6 is a membrane protein tethered to the ER by BiP [3]. During ER stress, ATF6 undergoes cleavage to its active transcription factor form in the golgi [3]. Further work is needed to determine if SKN-1/Nrf proteins at the ER have a similar fate.

Coordination between SKN-1/Nrf proteins and the UPR has been evolutionarily conserved and, therefore, may be fundamentally important to cell homeostasis. Direct regulation of the UPR implies that the ER may be able to prepare for protein damage under harsh conditions detected by SKN-1/Nrf family members. It may also ensure that redox homeostasis in the cytosol is compatible with redox-dependent protein processing in the ER. The activity of SKN-1 also responds to changes in the nucleolus [17], the proteasomes [18], nutrient signaling [5]–[6], and protein translation [19]. Therefore, an extremely complex network of signals likely converges on SKN-1 to ensure that redox status and detoxification activity are compatible with a number of cellular processes. Deciphering this signaling network will provide mechanistic insights into how a single transcription factor is influenced by multiple signals. As we continue to refine our understanding of cellular stress responses and their roles in disease and aging, it will be increasingly important to investigate how different pathways coordinate responses to optimize homeostasis, avoid incompatibilities, and mitigate competition for common substrates.
